# Restorative justice and the role of community in *All Your Faces*: a film analysis

**DOI:** 10.3389/fsoc.2026.1947491

**Published:** 2026-09-10

**Authors:** Alessandra Minissale, Raffaella Sette

**Affiliations:** 1 Department of Sociology, Uppsala University, Uppsala, Sweden; 2 IGSG-CNR, Bologna, Italy; 3 Department of Sociology and Business Law, Alma Mater Studiorum, Bologna University, Bologna, Italy

**Keywords:** circle, communities of care, community, film analysis, prison, restorative justice, victim-offender mediation

## Abstract

This article examines the representation of community within restorative justice (RJ) in the French film *All Your Faces* (*Je verrai toujours vos visages*, 2023), addressing central debates within RJ scholarship concerning the meaning, place, and function of community in restorative processes. By analysing the film’s verbal and visual dimensions, the article examines how community is represented and enacted through relationships, interactions, spatial arrangements, and restorative practices. The analysis identifies three forms of community: prison as a space in which multiple communities intersect; the participatory community that emerges within the restorative circle; and communities of care that support victims and offenders throughout the restorative process. Across these dimensions, *All Your Faces* portrays community as an evolving social process rather than a fixed category of participants. Community is progressively produced through ritualized interaction, storytelling, humor, collective vulnerability, recognition, and practices of care. These findings shift attention from who constitutes the community in RJ to how community is enacted through restorative practices and relationships. The article contributes to interdisciplinary scholarships at the intersection of sociology, law, criminology, and victimology by conceptualizing community as a relational and institutional accomplishment. It also demonstrates the value of film as a site for examining how restorative justice practices and values are culturally imagined, interpreted, and communicated to wider publics.

## 1 Introduction

This article offers a sociological film analysis of the French film *All Your Faces* (*Je verrai toujours vos visages*, 2023), directed by Jeanne Herry. Following Morin’s (2016) argument that cinema and sociology are deeply interconnected, films can be understood as a medium through which reality and imagination are refracted, providing a valuable lens for exploring the social representations of legal and social phenomena (Curti and Fornari, 2018). *All Your Faces* offers a nuanced cinematic portrayal of restorative justice (henceforth RJ) and, as such, participates in the construction of public understandings of justice, social relationships, and responses to harm. This cultural function is further significant given that films addressing victimization, victims’ needs, and RJ have been incorporated into RJ programs within prisons as educational and reflective tools (Van Camp and Wemmers, 2013; Robert and Peters, 2003). Through multiple, intersecting life trajectories, the film illustrates the diversity of RJ practices while foregrounding their emotional, relational, and social dimensions.

Specifically, the film^1^ develops two parallel storylines. The first follows a series of weekly in-prison circles between victims and offenders of robberies and home invasions, participated also by two representatives of the local community. As the meetings unfold, their individual stories, each portrayed with considerable emotional depth, gradually emerge, the relationships between participants evolve, and new forms of mutual understanding and social connection begin to develop. The second storyline centers on a victim of rape who initiates a RJ program to meet her stepbrother, the perpetrator. Interwoven throughout the film, these narratives portray different RJ practices (Loncan, 2024), demonstrating both the diversity of restorative interventions and their capacity to reshape relationships, focusing on the emotional, relational, and social dimensions of responding to harm.

The film’s title derives from a statement made by one of the incarcerated participants during the final restorative circle. Responding to one of the victims’ questions about whether he might return to crime after his release, the offender replies that he could not do it again, because he would see all their faces. The phrase encapsulates the film’s portrayal of RJ as a process creating enduring forms of recognition that reshape relationships and gradually foster a sense of community among everyone involved in the restorative process. In this article, we focus precisely on this aspect, analyzing how the film represents the role of community within RJ processes. Community has long been considered a foundational component of RJ (Braithwaite, 1999; Zehr, 2014), yet both its meaning and practical implementation remain contested (Rosenblatt, 2014; Rossner and Bruce, 2016). While RJ frameworks emphasize repairing harm through dialogue among those affected by wrongdoing, including victims, offenders, and members of the community, scholars have highlighted that the concept of community is often invoked in abstract or idealized terms (Willis, 2016; Rosenblatt, 2014).

By analyzing *All Your Faces* as a cultural representation of RJ, we explore how the film constructs and visualizes community within restorative encounters. The analysis focuses on three forms of community depicted throughout the film: prison as a space in which multiple communities intersect; the participatory community that emerges within the circle; and the communities of care supporting victims and offenders throughout the process. These dimensions reflect central debates within RJ scholarship concerning the place and function of community in RJ programs (Lupo et al., 2026). We show that *All Your Faces* depicts community as a social process rather than a category of participants. The sense of community is continually produced through ritualized interaction, storytelling, humor, collective vulnerability, and caring work. This shifts the question from who constitutes the community to how community is enacted through RJ. In this way, the article contributes to interdisciplinary scholarship at the intersection of sociology, law, criminology and victimology, demonstrating how film can serve as a valuable site for examining how RJ practices and values are imagined, interpreted, and communicated to wider publics. Accordingly, this article asks: How is community represented in a cinematic portrayal of RJ, and what does this representation reveal about the sociological meaning of community within restorative practices?

## 2 The role of community in restorative justice

RJ emerged as a response to the perceived limitations of conventional retributive criminal justice system, particularly their focus on punishment and legal procedure instead of the relational consequences of harm (Braithwaite, 1989). Building on the classical theory of Beccaria (1769), retributive justice is generally conceptualized as the obligation of offenders to repay a debt owed to society, grounded in the belief that punishment and the resulting suffering can discourage future criminal behavior. Within this framework, the primary actors are the offender and the State, which represent the abstract and symbolic political community that punishes on behalf of society (Duff, 2000). By contrast, the victim “for most of the proceedings is pushed completely out of the arena, reduced to the triggerer-off of the whole thing. Victims are some sort of double loser; first, vis-a-vis the offender, but secondly and often in a more crippling manner by being denied rights to full participation in what might have been one of the more important ritual encounters in life” (Christie, 1977, p. 3).

Opposite to that, RJ processes are commonly described as involving three key groups: victims, offenders, and community members. Community participation is understood to provide broader social recognition of harm and to support processes of accountability, reintegration, and repair. The conceptual shift from retributive to restorative justice can be summarized along three key dimensions, as illustrated in Table 1.

**Table 1:** Key conceptual differences between retributive and restorative justice.

Dimension	Retributive justice (Duff, 2000)	Restorative justice (Zehr, 2014)
Understanding of crime	Violation of law	Harm to people and relationship
Harmed party	State/political community	Victim and community
Response to wrongdoing	Punishment	Repair and reconstruction of interpersonal community relations

At the core of RJ lies the idea of “making things right”. Justice and healing are pursued through processes that are co-constructed by victims, offenders, and community members, thereby fostering reconciliation at both individual and collective levels (Zehr, 2014). In this sense, RJ is fundamentally relational, as its ethos emphasizes the idea of repairing and maintaining healthy relationships among offenders, victims, and other community members (Scholl and Townsend, 2024). Within this framework, community is conceived of an active participant in responding to conflict and crime, and as such as a distinctive stakeholder (Maglione, 2017; Asadullah and Morrison, 2021).

Community participation in RJ is multifaceted (McCold, 2004). It may involve members of victims’ and offenders’ immediate networks, known as “micro-community” (Rossner and Bruce, 2016), trained volunteers representing the wider community, known as “macro-community” (Dhami and Joy, 2007), or community members who support reparation and reintegration beyond the restorative encounter (Bazemore and Karp, 2004). Regardless of the format, community participation is seen as essential to strengthening informal mechanisms of social control, problem-solving capacities and social cohesion (Caselli, 2024). The United Nations Office on Drugs and Crime (UNODOC) (2020) similarly frames community involvement in restorative processes not only as an object of restorative policies, but as part of a participatory paradigm aimed at reducing social alarm around crime, and at re-embedding responses to harm within the social fabric of affected communities.

However, the concept of community within RJ has been widely debated particularly because its mobilization presupposes the identification of concrete individuals and groups who are both affected by conflict and capable of participating in its resolution (Caselli, 2024). As noted by Johnstone (2014), RJ discourses often rely on an idealized conception of community as cohesive, stable, and morally engaged in addressing wrongdoing. In practice, however, communities are complex social formations characterized by diversity, conflict, and unequal power relations. As a result, the role of community within RJ processes is neither uniform nor straightforward, and in need of conceptual as well as practical clarification (Pavlich, 2004; Maglione, 2017).

The definition of what constitutes a community, and who should represent it, also depends on the objectives that RJ is intended to achieve (Kirkwood, 2022). From this perspective, the purist approach maintains that RJ should be limited to situations involving criminal offenses, as opposed to other forms of harm. Accordingly, the role of the community may remain relatively undefined, as the restorative process is primarily centered on the relationship between victims and offenders. By contrast, the maximalist approach views RJ as an opportunity to facilitate communication between those who have suffered harm—not necessarily resulting from a criminal offense—and those responsible for it. From this perspective, it may be appropriate to identify, on a case-by-case basis, members of the community who have a legitimate interest in participating both in repairing the consequences of the conflict and in supporting the parties’ reintegration into the community. Finally, an even broader approach conceptualizes RJ as a social movement, advocating the use of restorative practices across a wide range of settings and, more fundamentally, seeking to transform the ways in which people relate to one another. These latter two approaches can be situated within the broader paradigm of community justice (Clear et al., 2010).

Scholars have increasingly focused on how community participation is enacted in restorative practices. Rosenblatt (2014), for instance, highlights the tension between the normative ideal of community participation and the realities of contemporary justice systems. Her work shows that while RJ frameworks frequently emphasize community involvement, in practice this participation is often mediated through professionals or institutional actors rather than local communities themselves. van Staden and Slump (2023) further point to the practical difficulties of involving community representatives due to lack of information about the possibility and (dis)advantages of RJ. As a result, community representatives may be reduced “to a small number of ‘core volunteers’ becoming ‘quasi-professionals’” (Crawford, 2002). Similarly, Rossner and Bruce (2016) identify a process of quasi-professionalization whereby community roles are increasingly occupied by trained facilitators or volunteers rather than by organically involved community members. Willis (2016) attempts to establish conceptual clarification by distinguishing three practical forms of community: community as participation in restorative processes, community as networks of social support surrounding participants, and community as the broader social context affected by crime. This distinction provides a useful framework for analyzing how community is depicted and enacted in RJ.

Building on the theoretical and empirical perspectives outlined above, in this article we examine how the film *All Your Faces* portrays different dimensions of community through its narrative and visual representation of restorative encounters.

## 3 Methods: film analysis as a means to sociological inquiry

The present article adopts qualitative film analysis as a method to examine how the film *All Your Faces* represents community within RJ. Film analysis has increasingly been recognized as a valuable methodological tool within qualitative social research, particularly for examining cultural representations of social institutions and practices (Teixeira et al., 2021). Films can be understood as cultural representations that both reflect and shape social understandings of institutions, relationships, and moral values (Mikos, 2013). Following Rose (2016), visual materials are seen as cultural constructions through which meanings are produced and communicated, and social phenomena are interpreted and made intelligible. Film analysis is therefore particularly suited to socio-legal research because it enables scholars to examine how legal institutions, and RJ more specifically, are made meaningful within wider cultural imaginaries.

*All Your Faces* provides an especially rich case for such analysis because its narrative focuses on the interactions unfolding before, during, and after restorative encounters. The film provides an opportunity to examine how RJ constructs relationships between victims, offenders, facilitators and community members, and particularly how different forms of community emerge throughout the restorative process. The film’s significance for the RJ field is also reflected in its inclusion among the films and documentaries collected by the European Forum for Restorative Justice (EFRJ) and its selection by the EFRJ for screening and discussion during the International Restorative Justice Week in 2023.^2^

Our empirical process consisted in watching the film^3^ first without interruptions, gaining an overall understanding of the narrative structure, character development and emotional trajectory, and a second time with systematic pauses, to identify material relevant to the research question. Specifically, we selected scenes and dialogues portraying the restorative encounters themselves, as well as scenes and dialogues that contributed to the film’s representation of community, including moments in which (a) relationships among victims, offenders, community representatives, facilitators, family members, and intimate partners were established or transformed; (b) participants expressed or negotiated belonging, recognition, care, vulnerability, or social connection; (c) the spatial and procedural organization of restorative encounters transformed interactions and relationships among participants. Our selection process therefore extended beyond explicit references to “community” to include the relational and institutional practices through which community was represented and enacted in the film.

Both authors independently recorded analytical notes. These were subsequently compared and discussed jointly to refine the analytical categories and identify recurring themes across the film. The analysis combined attention to verbal and visual dimensions of the film. Following Mikos (2013), we understand meaning as emerging from the interaction between narrative, visual composition and emotional performance. We analyzed dialogues to understand how participants describe harm, fear, responsibility, care, and belonging. Visual elements, such as organization of space, camera framing, bodily positioning, movement and silence, provided access to the ways through which community is symbolically constructed. While attention to verbal and visual dimensions is consistent with qualitative approaches to film analysis (Mikos, 2013; Rose, 2016), the specific elements examined were selected for their relevance to our research question and refined through the abductive analytical process described above. Accordingly, cinematic features unrelated to the representation of community, such as lighting, sound, and editing, were not systematically analyzed.

Our analysis followed an abductive approach, moving back and forth between theoretically informed concepts concerning community participation (Rosenblatt, 2014; Rossner and Bruce, 2016; Willis, 2016), communities of care (Willis, 2016; Bolivar, 2012), and RJ in prison settings (Johnstone, 2014; Robert and Peters, 2003) and themes emerging from repeated engagement with the film, which allowed additional dimensions, such as spatial organization and informal interaction, to become analytically salient. The abductive process resulted in three main analytical categories, which structure the findings presented in the current article: prison as a space for intersecting multiple communities, community within the restorative circle, and communities of care. In the analysis, we include short extracts of dialogue to illustrate how the film constructs understandings of RJ and community.

## 4 Prison as a space for intersecting communities

At first glance, prison appears an unlikely setting for a story about community. Designed to separate offenders from society, prisons are conventionally associated with confinement, exclusion and the enforcement of legal punishment (Di Gennaro et al., 1997). The film *All Your Faces* presents prison as the institutional setting in which restorative encounters become possible and where individuals who would otherwise remain separated by crime and criminal justice are temporarily brought together.

This inversion becomes apparent from the opening sequences. The central narrative space of the film is the prison circle facilitated by Fanny and Michel. Participants include three non-specific victims (Grégoire, Nawelle, and Sabine), three incarcerated offenders (Nassim, Thomas, and Issa), and two representatives of the local community (Yvette and Cyril). This configuration of the circle as portrayed in the film also deserves attention. Restorative programmes vary considerably in their organization and participant composition across institutional and national contexts (Robert and Peters, 2003). The collective format (victims and offenders together) depicted in *All Your Faces* should therefore be understood as a specific institutional arrangement rather than as representative of prison-based restorative practice generally. Such variation also illustrates how restorative principles are translated into different organizational forms as they become institutionalized within existing justice system.

Before participants reach the room designated for the circle, the camera follows them through security gates, metal detectors, locked doors, and long prison corridors. As prison visitors, victims, facilitators and community representatives leave their personal belongings, wear identification badges and are escorted through a succession of controlled spaces. This visual language of imprisonment (Goffman, 1961), with emphasis on surveillance, restriction and separation, clashes with the intimate setting of the brightly colored room of the restorative encounter, organized around a circle of chairs, paintings, coffee, and homemade cakes. The contrast between the prison architecture and the restorative room visually communicates that a different social order operates within the same institutional setting. Within the punitive institutional setting of the prison, the restorative programme creates a carefully structured space in which emotions, interactions, and relationships are regulated according to restorative principles. In this sense, while participants remain physically inside prison, the restorative circle temporarily reconfigures that setting into a different symbolic space governed by listening, dialogue, and mutual recognition. The restorative encounter transforms the institution into a place where reflection becomes possible and where the boundaries between punishment and repair are continuously negotiated. In doing so, the film visually captures what Dubois (2008) describes as the coexistence of punitive and restorative models of justice within the same institutional setting.

This representation reflects a growing body of scholarship examining RJ in prisons. While RJ was initially conceived primarily as an alternative to incarceration, scholars have increasingly argued that prisons themselves can become important sites for restorative practices (Johnstone, 2014). Empirical studies further demonstrate that prison-based restorative justice programs can encourage offenders to recognize the human consequences of their actions, develop empathy towards victims, strengthen connections with the wider community, and assume greater personal and social responsibility (Rees and Hobson, 2021; Richner et al., 2023; Duryea et al., 2024; Mannone, 2026). These programs seek to complement punishment in prison by creating opportunities for moral repair and relational reconstruction within custodial settings. The coexistence of these institutional logics becomes particularly visible when Issa (one of the offenders) is unable to attend one of the circles:

Fanny: We’ve got a problem. Issa will not be with us today. He cannot make it. We’re sorry,

Grégoire: Why?

Fanny: We have no details. He’s in a disciplinary block. The question we have for you is: do we hold the session without him?

Grégoire: Why’s he in the block? Your team blocked him? He got in trouble?

Fanny: No, I don’t think the prison’s being obstructive. We don’t know what happened.

Grégoire: Sorry, I just don’t get it. These meetings matter.

Fanny: Of course.

Grégoire: Months preparing, committed to this schedule…

Fanny: Apparently Issa clashed with some guards. It just takes one incident, a cell search that goes wrong.

The exchange illustrates how the restorative space remains embedded within, and dependent upon, the institutional order of the prison. Although the restorative program creates its own commitments, relationships, and temporal structure, participation remains subject to the rules and disciplinary decisions of the custodial institution. Issa’s absence reveals the practical tension between these coexisting institutional logics, as the commitments generated within the restorative process remain subordinate to the prison’s disciplinary authority. At the same time, Grégoire’s frustration is significant because, as a victim, he expresses concern about the absence of one of the offenders, suggesting that repeated participation has already begun to generate expectations of mutual commitment and responsibility among members of the circle. Prison therefore performs a function that extends beyond serving as the backdrop to the narrative. It becomes the institutional threshold through which different communities, which are normally kept apart by crime, punishment and social stigma, can encounter one another under carefully organized conditions.

The introduction of the community representatives, Yvette and Cyril, is equally revealing. At the first meeting, Fanny (facilitator) merely presents them by explaining that *“they’ll be present at every meeting”* before immediately moving on to the practical organization of the program, without further justification regarding why they are there or what function they perform. While this apparent lack of explanation may reflect the structure of RJ programs, where victims and offenders undergo a series of individual preparatory meetings during which the aims, procedures, and participants are discussed, the film nonetheless presents community participation as an ordinary and self-evident feature of RJ. This reflects core concerns within RJ scholarship, which questions the importance of clarifying who constitutes “the community,” who can legitimately represent it, and how community representatives acquire their legitimacy (Johnstone, 2014; Maglione, 2017; Rosenblatt, 2014). The film *All Your Faces* leaves these questions open to interpretation as community representatives appear an institutionalized and unquestioned component of the process. The absence of further explanation may also reflect the French restorative justice framework on which the film is based. French legislation does not specify who should constitute ‘the community’ or how community representatives should be selected. Hence, the film may mirror this institutional ambiguity by presenting Yvette and Cyril’s participation as a routine element of the restorative process.

The role of community in the movie is further complicated during Cyril’s self-introduction, as he initially states that his presence demonstrates that “*society does care about you* [the circle’s participants],” before immediately correcting himself to say that society *“takes an interest in you”*. This moment is noteworthy because whereas *care* evokes emotional closeness, compassion, and personal commitment, *taking an interest* suggests a more measured and emotionally restrained form of engagement. In doing so, the film reflects the increasingly institutionalized character of community participation identified in RJ literature, where community volunteers are seen as occupying an intermediate position between ordinary citizens and professional facilitators, becoming quasi-professionals (Rossner and Bruce, 2016). Specifically, Cyril embodies a form of civic engagement in which community representatives are expected to offer recognition, support, and symbolic inclusion while maintaining the emotional boundaries required for RJ facilitators. His self-correction thus illustrates the ambiguous emotion norms (Hochschild, 1983) governing this role. Community representatives are expected to convey concern and human solidarity without becoming too emotionally involved, continuously negotiating the boundaries between caring and institutional neutrality. Their participation requires balancing emotional involvement with emotional restraint.

Interestingly, as they first enter prison, victims begin forming a community of their own before meeting the offenders. While waiting for the circle to be complete, Grégoire explains to Sabine and Nawelle that, despite therapy, *“the wound is still there”* and he has stopped talking about the experience because “*nobody understands*.” Nawelle immediately recognizes this experience, replying that she no longer speaks about the robbery, not even with her husband or children. In this way, victims recognize a common sense of emotional isolation and estrangement from their everyday social worlds, in which they feel unable to communicate the continuing impact of crime. This shared emotional isolation and estrangement seem to create opportunities for “empathic resonance,” a process in which expressed similar emotions facilitate the attuning with each other’s feelings (Abramson and Moore, 2002).

Equally revealing is the fact that emotional convergence is not presented as a prerequisite for belonging. During their conversation before the circle, Nawelle openly describes to Sabine and Grégoire that she sees the program as “*a battle*” and anticipates confronting participants whom she believes *“have no heart”*. When she asks Grégoire whether he is equally angry, he quietly responds that he is not sure. This emotional mismatch is accepted without hesitation, as Nawelle simply smiles and offers to fetch him a coffee.

Sabine, instead, initially mistakes Grégoire for one of the offenders. This misunderstanding is interesting as it highlights how, within the prison setting, the identities of victims and offenders may be confusing as they are not immediately visible but must be discovered through interaction. Sabine apologizes and admits being relieved that he is part of the group, remarking that his presence makes her feel safer because *“he’ll protect us”*. Sabine’s relief upon learning that Grégoire is a fellow victim suggests that participants begin to orient themselves toward one another by seeking sources of safety and solidarity within the group. Her remark, together with Nawelle prior interaction with Grégoire, draws attention to the way participants begin constructing a shared identity as victims even though they are complete strangers. By showing that community emerges despite divergent emotional responses to victimization and through a search for solidarity, the film suggests that victims’ sense of belonging to a shared community relies on the willingness to recognize and accommodate one another’s different ways of living with harm and to support and being supported by one another. In this way, victims build a community based on their shared sense of vulnerability (van Stokkom, 2002).

To recapitulate, rather than presenting prison and RJ as mutually exclusive institutional logics, the film depicts their coexistence within the same legal order, illustrating how different conceptions of justice may be institutionally articulated and negotiated (Robert and Peters, 2003). The restorative program creates a space within the prison, even before the restorative circle formally begins, in which victims develop a sense of community. Once participants enter the restorative circle, they construct a wider community that comprises offenders. Ritualized interaction, storytelling, humor, and collective vulnerability foster mutual recognition and gradually transform a gathering of strangers into a participatory community, as we shall see in the following section.

## 5 Community within the restorative circle

This section examines how *All Your Faces* portrays the restorative circle as a site of community-building through repeated interactions that allow victims and offenders to move from social and emotional distance toward recognition, familiarity, and solidarity.

The film departs from conventional representations of community as a pre-existing social entity waiting to receive offenders back into society, as exemplified by Nassim (one of the offenders), who emphasizes that his participation to the circle is something he does to *“look good”* to society once he gets out of prison. The film then portrays community as an interactional accomplishment that gradually emerges through ritual and emotional vulnerability. The community created within the circle may be seen as prodromal to offenders’ reintegration, meaning “the incorporation of the offender into a community-based prosocial moral order” (Van Ness and Strong, 2015, p. 39).

During the opening meeting, participants enter the room hesitantly, avoiding prolonged eye contact and with long silences punctuating their early conversations. The camera repeatedly focuses on nervous hands, lowered gazes, and restrained facial expressions of both victims and offenders. The circle-community is therefore initially represented through its absence. Although participants occupy the same physical space, they remain socially and emotionally distant, united only by the consequences of crime. This initial discomfort reflects one of the central tensions identified within RJ scholarship. As Rosenblatt (2014) observes, community cannot simply be assumed to exist because individuals are brought together within a restorative program. Instead, community becomes one of the outcomes of the restorative process. The film *All Your Faces* visualizes this process by depicting how relationships are gradually built through repeated, ritualized interaction and storytelling. Specifically, the circle works as a ritualized social space with procedural rules, such as speaking being regulated by a baton-like talking piece creating a sense of equality among participants. Such ritual contributes to transforming an otherwise unfamiliar group of strangers into what Rossner (2013) describes as an emotionally synchronized interaction. Participants narrate the ways in which crime has reshaped their lives. Victims speak about fear, shame, anger and loss of confidence, while offenders describe family histories, addiction, prison, and the circumstances surrounding their offending. This structure allows for intertwining narratives of accountability and harm to develop and for participants to start shifting their emotional understanding of each other’s worlds (Rossner, 2013).

One of the earliest moments illustrating this shift occurs when Nawelle describes the armed robbery she experienced while working in a supermarket. She recounts the continuing emotional consequences of the event, such as the inability to feel safe, persistent anxiety, and the lasting disruption of everyday life, foregrounding the emotional aftermath of crimes that criminal proceedings rarely make visible (Zehr, 2014). Equally significant is the way in which the camera represents listening. As Nawelle talks, the camera frequently shifts away from the speaker towards those receiving the story. Close-ups linger on subtle changes in facial expression, moments of discomfort and gestures of recognition. This cinematographic choice reflects the importance accorded to voice and participation in RJ, whereby those affected by harm are given the opportunity to articulate their experiences and have them acknowledged by others (Zehr, 2014). The audience is invited to witness the listeners becoming emotionally implicated in another person’s experience.

The same dynamic emerges when Grégoire recounts the violent home invasion that profoundly affected his family. He explains that the physical assault was only one dimension of harm. More enduring was the fear experienced by his young daughter and his own sense of failure for being unable to protect her. As he speaks, the camera again privileges the reactions of those around the circle. Thomas (one of the offenders) lowers his gaze like Nassim (another offender), who appears visibly unsettled. Sabine (one of the victims) listens intently before quietly acknowledging the familiarity of her emotions. Restoration thus begins to emerge through collective witnessing while individual storytelling progresses. Harm is progressively transformed from an individual experience into a collectively recognized reality (van Stokkom, 2002). Through this process, responsibility itself begins to shift. Offenders move attention from abstract legal categories (e.g., crime, victim, convict, inmate) to concrete human experiences, while victims increasingly encounter offenders as complex individuals. Community therefore develops through reciprocal recognition.

This transformation becomes particularly visible in the evolving responses of the offenders. At the beginning of the program, Nassim openly questions why victims *“don’t move on”*. As the meetings continue, Nassim gradually abandons defensive explanations and begins acknowledging dimensions of victimization that he had previously failed to consider. Accountability therefore emerges through sustained exposure to the emotional realities of others. A sense of community develops as individuals become increasingly capable of recognizing the legitimacy of one another’s experiences. This portrayal resonates with a growing body of RJ scholarship that challenges understandings of community as a cohesive moral entity and instead emphasizes how forms of community are actively constructed through participation in restorative programmes (Willis, 2016; Rosenblatt, 2014). As Maglione (2017) observes, community is frequently described as a cohesive network characterized by shared understandings, common values and emotional connectedness. The film *All Your Faces* portrays these processes as gradual accomplishments of the restorative process itself, where membership is progressively established through repeated encounters and integration emerges as victims and offenders start recognizing one another as legitimate interlocutors.

This reciprocal recognition becomes particularly visible when Sabine (one of the victims) breaks down while describing how fear has restricted her life and separated her from her children and grandchildren. As she cries and describes herself as “pathetic,” support comes from across the circle:

Issa [stands up, approaches Sabine and kneels beside her]: It’s okay, it’s not too late [reassuring tone, concerned face].

Grégoire [moves towards Sabine]: It’s not too late, not at all. I talk to myself. You’re not pathetic.

Thomas: You weren’t scared to meet three dickheads like us.

Sabine: I was scared.

Thomas: Even more respect for that.

Nawelle [places a hand on Sabine’s leg]: He’s right. You’re so brave.

The scene makes the emerging community visible through both verbal and embodied forms of solidarity. Support crosses the victim-offender divide as Issa physically moves across the circle and kneels beside Sabine, while Grégoire and Nawelle similarly reduce the physical distance between participants. Thomas’s intervention combines recognition and humor, reframing Sabine’s fear as courage. Solidarity emerges through participants’ responses to their mutual vulnerabilities.

Another important sign of community-building within the circle is the fact that, as the program progresses, the interactions taking place within the restorative circle increasingly extend beyond the formal discussion of crime. Participants begin engaging as ordinary individuals with shared concerns, interests, and aspirations. This transformation is visible in the changing dynamics of the discussions themselves. Whereas the first meetings are characterized by hesitation, long silences, and strict adherence to procedural rules, later encounters become noticeably more conversational. Participants increasingly respond directly to one another without waiting for facilitator prompts, they challenge their mutual assumptions, ask spontaneous questions, and occasionally interrupt with humor or expressions of encouragement. The talking piece, initially central to regulating dialogue, is gradually abandoned at the request of one of the victims, Nawelle, who suggests that the group no longer needs it because they have learnt the rules. This shift is particularly revealing as it signals that the procedural structure that initially organized interaction gradually becomes internalized, allowing participants to engage in more spontaneous forms of conversation. Meetings thus acquire the rhythm of an ordinary conversation among people who have become familiar with one another.

An interesting exchange concerns Issa’s childhood (another offender). During one session, he recounts the violence he experienced at the hands of his father but initially rejects the suggestion that he himself had been abused, minimizing experiences that included repeated beatings and physical punishment. Specifically, Issa, echoed by Nassim, diminishes slapping to non-violent behavior, opposite to hitting:

Issa […] Slapping, not hitting, like, hold still.

Grégoire: slapping is hitting.

Issa: No, hitting is with guns, or feet and fits. That’s just… [looks at Nassim in search of help]. My mom used to slap me. Dad hit me.

Nassim: That’s right. My dad slapped me, my uncle hit me. Like for Sabine… the guy hit you. It’s out of line.

Eventually, Issa himself begins reconsidering his earlier understanding of his childhood. What makes this scene particularly significant is the way recognition emerges collectively as participants help one another reinterpret their own biographies through dialogue, demonstrating that the restorative circle functions as a space in which new understandings of personal experiences become possible. The sense of collective vulnerability (van Stokkom, 2002) that had united victims within a distinct community early in the movie has now expanded by involving offenders too.

The restorative circle is equally transformed through humor. RJ scholarship has often focused on empathy, remorse, and emotional disclosure as key ingredients for successful rituals within RJ (Rossner, 2013). The film demonstrates that humor also plays a central role in creating community. It appears cautiously at first, before becoming an increasingly frequent feature of the meetings. Participants tease one another, joke about everyday life, and laugh collectively, signaling a growing ease within the group. One example occurs when Issa reassures Sabine after her emotional disclosure, before joking about her struggles with digital technology, prompting laughter throughout the room. Far from undermining the seriousness of the discussion, humor functions as a mechanism through which emotional intensity becomes bearable and relationships become more ordinary (Scarduzio, 2011).

Perhaps even more revealing are the interactions occurring outside formal meetings. During coffee breaks participants smoke together, share homemade cakes prepared by community volunteers, discuss recipes, gardening, employment, and family life. These scenes occupy a central place in the film’s representation of RJ as they are indicative of community-building, with the boundaries separating victims, offenders, facilitators, and community representatives becoming increasingly blurred.

Another significant illustration of this transformation concerns Thomas’s (one of the offenders) worry about life after release. This conversation turns towards employment, and Thomas openly admits that he may return to prison because he sees few realistic opportunities outside. His uncertainty prompts a response from Grégoire (one of the victims), who immediately offers practical assistance.

Grégoire: [addressing Thomas] What are you talking about? Interviews, filling out forms is tough? Life here is tough. I don’t get it. The crap yard, the rock-hard beds, that’s tough. What are you talking about? I’ll sort your forms, piece of cake. No shit. You call me, I’m there. Go back to jail, I’ll smack you.

Thomas nods and smiles, looking relieved.

The significance of this exchange, with a victim voluntarily assuming practical responsibility for supporting an offender’s reintegration into society, draws attention to the fact that community here becomes tangible through concrete acts of solidarity and care, as we shall develop further in the next section.

This movement from emotional dialogue to practical assistance culminates during the final gathering. At this point, Sabine (one of the victims) reveals that she wants to repair Thomas’s favorite shirt, which he wore at all five meetings, and even purchased him another one because she knew how much he liked it. This gesture carries particular significance because Sabine has explained that, since the home invasion, she had been unable to go shopping, remaining confined to her home out of fear that she might be attacked again. Purchasing a shirt for Thomas therefore symbolizes an act of care toward one of the offenders but also her gradual re-engagement with everyday life beyond the trauma. Others exchange telephone numbers and discuss maintaining contact after the program. These gestures demonstrate that restoration is accomplished through ordinary acts of reciprocity that characterize everyday social relationships, culminating in physical, affectionate contacts (Karstedt and Rossner, 2019).

The role of community representatives, Yvette and Cyril, during the circle, further contributes to debate around community participation and quasi-professionalization (Rossner and Bruce, 2016). Yvette and Cyril occupy a marginal position, remains often silent, and appear passive. They quietly sustain the emotional atmosphere of the program by welcoming participants, preparing refreshments, sharing meals, and offering informal support during breaks. While this may contribute to creating relational conditions that allow difficult conversations to unfold safely, their passive role during the circle-conversations may suggest an increasing institutionalization of community participation, which risks becoming merely symbolic (Rossner and Bruce, 2016).

In summary, the film *All Your Faces* suggests that community within the circle is built through ritual, storytelling, humor, informal interactions, and caring work. The signs of a new participatory community emerging from and within the circle can be traced in reciprocal emotional understanding, collective vulnerability, ease, and solidarity. These practices, however, do not conclude with the restorative circle itself. Instead, they extend beyond the formal meetings into broader networks of support and responsibility, to which the analysis now turns.

## 6 Communities of care

The film *All Your Faces* portrays RJ as embedded within wider networks of care that prepare participants for dialogue, accompany them throughout the process, and continue after the meetings have concluded. Community extends through a constellation of relationships involving facilitators, psychologists, volunteers, partners, and family members. These actors make restorative encounters possible by providing emotional as well as practical support. This understanding resonates with recent RJ literature, which has progressively moved beyond conceiving community simply as a body of citizens participating in restorative encounters (Willis, 2016). Throughout *All Your Faces*, community emerges as a series of caring practices through which individuals enable one another to engage with difficult emotion work.

A significant example is provided by Judith, the mediator supporting Chloé (the victim of rape committed by her stepbrother Benjamin) throughout her restorative journey. Judith’s work occurs largely outside the restorative encounter itself. She prepares Chloé, as well as Benjamin, who had been convicted and incarcerated, for the possibility of dialogue within the framework of a victim-offender mediation (henceforth VOM). Importantly, Chloé does not seek mediation to reconcile with Benjamin. Having learnt that he has returned to living in the same area, she wishes to confront him, to establish practical arrangements enabling her to never cross his path in the future.

Judith helps Chloé reflect on her expectations, fears, and emotional boundaries before any meeting takes place, but the mediator often appears cold and distant, to the point that Chloé explodes in anger by shouting when Judith prepares her for the possibility that Benjamin might want Chloé to ask for his forgiveness:

Judith: You must ask yourself. And reflect on the issue of forgiveness generally. Forgiving someone, asking to be forgiven.

Chloé [pauses, looks straight at Judith]: He wants me to ask his forgiveness? Right? He wants me to ask for his forgiveness [accelerates pace]. What do you think? You understand that? What would you say to that? After all this, and he still says that? What are you doing here? [starts screaming] You prep me, not him! I slog for months, digging it all up… I’ll never ask him to forgive me! He’s mental. You told him he’s mental? [keeps screaming].

Judith [calm, detached]: that’s your right. I’ll prepare your brother for the possibility you won’t do it. Even if you refuse… can you cope with it? Can you handle… him asking you to say sorry? You don’t have to answer now. Take your time.

Chloe: Yes, I know what you’re going to say. I’ll think it over, talk with my therapist.Judith: That’s really important.

Chloe: Yes… I get the rules and all. Sometimes it really hurts that you protect us both equally. I understand.

Judith’s composed and calm reaction to Chloé’s emotional outburst illustrates what recent ethnographic research has identified as one of the defining features of RJ facilitation: the simultaneous performance of care (Van Camp and Wemmers, 2013) equidistance (Mannozzi, 2013), and emotion work (Hochschild, 1983). Throughout the movie, Judith continually monitors the emotional safety of both participants while maintaining an impartial and neutral position. As Nascimento (2025) shows, facilitators accomplish neutrality and impartiality through continuous emotion work and reflective practice, regulating both their own emotions and those of participants to sustain the emotional conditions necessary for restorative dialogue. Judith continually negotiates the tension between care and impartiality that characterizes restorative practice, further engaging in continuous debriefing with her trainer. Facilitators are thus represented as belonging to the community of care supporting victims and offenders, particularly in relation to how they seek to ensure the safety of the encounter during the preparatory work before the restorative process. Managing expectations is also presented as a key component of facilitators’ caring work. This representation resonates with Van Camp and Wemmers (2013), showing that preparatory meetings extend well beyond procedural preparation as facilitators address participants’ emotional needs, clarify their expectations, help formulate questions, and emotionally prepare participants for the encounter, thereby making change with RJ itself a source of care, support, and empowerment.

While professional facilitators and psychologists provide the institutional framework through which restorative encounters become possible, the film *All Your Faces* also demonstrates that RJ depends upon more intimate communities of care. These relationships become integral to RJ success by sustaining participants emotionally before, during and after the encounter. An example of this is Chloé’s partner, Mehdi. Unlike Judith, whose role is institutionally defined, Mehdi represents a form of ordinary care grounded in intimacy and daily life. The movie shows that Mehdi avoids openly influencing Chloé’s decision about whether to meet Benjamin. Behind closed doors, however, Mehdi meets Judith and passionately expresses his concerns for the restorative process potentially hurting Chloé. Despite these concerns, Mehdi ultimately supports Chloé’s choice. Significantly, while she meets Benjamin inside the mediation room, Mehdi waits quietly outside the building, ready to accompany her immediately afterwards. His role brings to the fore the ambivalent position of those closest to victims, who seek to protect them from further harm while simultaneously respecting their autonomy and supporting their restorative choices.

Once the encounter between Chloé and Benjamin takes place and the conversation commences, Chloé immediately assumes control while Benjamin’s responses remain short:

Judith: We agreed that Miss Delarme [Chloé] would speak first. Chloé, over to you. [Throughout the opening, Benjamin repeatedly looks at Chloé, while she avoids his gaze].

Chloé [looking at Benjamin for the first time]: We live in the same town, but I don’t want to bump into you. I want us to agree on things, and stick to them [pauses, Benjamin looks at her, mouth slightly open in a mix of surprise and unease, looks close to crying.] You plan to visit Mum’s grave?

Benjamin: Yes.

Chloé: Which day would you go?

Benjamin: At the weekend.

Chloé: The weekend, okay. I won’t ever go at weekends. Only during the week. And you never go during the week. Okay?

Benjamin (visibly surprised, his eyes becoming tearful): Okay.

[The discussion continues as Chloé allocates different days for visiting the cinema, restaurants, and other public places, while Benjamin quietly accepts each arrangement.]

By structuring the conversation according to her own priorities, Chloé reclaims the agency previously denied her through years of silence, family conflict, and criminal proceedings. Benjamin repeatedly looks towards her as the meeting begins, but Chloé avoids his gaze until Judith invites her to speak. From this point onward, Chloé looks directly at Benjamin until the end of the encounter. She remains steady and controlled while Benjamin appears fragile and emotional. Through this subtle choreography of gaze and speech, the film visually represents the recovery of agency and empowerment that the restorative process seeks to facilitate. Restoration emerges as Chloé’s renewed ability to inhabit her everyday community on terms that she herself defines.

Immediately after Chloé’s meeting with Benjamin, the film follows Chloé as she leaves the building and meets Mehdi. Without asking what happened or demanding explanations, he simply embraces her. In this way, the film portrays communities of care as essential for restoration and as extending beyond institutional figures, suggesting that restoration builds on the presence of caring individuals who accompany participants afterwards. This resonates with Bolivar’s (2012) victim-centered understanding of community, in which care is experienced primarily through supportive relationships rather than formal participation in restorative processes.

This attention to care beyond the formal encounter reflects a broader understanding of RJ increasingly recognized within the literature. As Willis (2016) argues, communities of care encompass those relationships that enable restorative practices to function, both for victims and offenders, even though they are rarely formally acknowledged within restorative programs themselves. The film equally complicates the notion that family naturally functions as a supportive community (Pavlich, 2004). Chloé’s wider family repeatedly struggles to acknowledge the seriousness of the abuse she experienced, illustrating how communities may simultaneously provide belonging and reproduce harm.

Turning to the role of communities of care within the circle, the film shows that caring gestures from victims to offenders are a sign of community building, as discussed in the previous section. The restorative program similarly reshapes relationships extending beyond direct victims and offenders. One particularly significant example concerns Nassim’s relationship with his sister. Reflecting on his participation in the program, he explains that listening to victims gradually allowed him to understand why his sister had distanced herself from him. Further prompted by Nawelle’s physical resemblance to his sister, Nassim decided to phone her, and she offered to let him live with her as soon as he got out of prison. The circle therefore helps transform Nassim’s interpretation of existing family relationships. Restoration extends outward from the encounter, enabling him to reconnect with someone who had long ceased to trust him. The film thus suggests that RJ may allow fragmented communities to be rebuilt and transformed into communities of care as a result of the restorative process.

## 7 Conclusion

This article examined how the film *All Your Faces* represents the role of community within RJ. The film portrays community as an institutional accomplishment, gradually produced through legally organized encounters, ritualized interaction, and practices of care. In doing so, it offers a sociological reflection on the ways RJ, a legally institutionalized response to crime (cf. Cotterrell, 1995; Pennisi, 2024), contributes to constructing forms of social relations, responsibility, and collective belonging. By analyzing the prison setting, the restorative circle, and the wider networks of care surrounding participants, the article has shown that the film offers a multilayered understanding of community that both reflects and extends current debates within RJ literature.

First, *All Your Faces* portrays restorative practices as coexisting with punitive institutions, transforming prison into a space where dialogue and moral reflection become possible. Prison becomes a space where different communities, victims, offenders, facilitators, and volunteers temporarily intersect. This representation resonates with scholarship arguing that RJ in prison can complement punishment by creating opportunities for accountability, empathy, and relational repair (Johnstone, 2014; Robert and Peters, 2003), while also highlighting the symbolic significance of bringing together individuals who would otherwise remain separated by crime and institutional boundaries.

Second, the film challenges idealized conceptions of community as a pre-existing collective characterized by cohesion, shared values, and emotional consensus. The first sense of community emerges even before the restorative circle begins, as victims recognize their respective experiences of emotional isolation and the enduring consequences of victimization. This initial bond is grounded in shared as well as diverging emotions, in victims’ willingness to acknowledge and accommodate their different ways of living with harm, and in their search for solidarity and support. Only subsequently does this victims’ community expand within the restorative circle to include offenders through ritualized and informal interactions, storytelling, humor, and collective vulnerability. Community therefore appears as the outcome of an evolving relational achievement, institutionalized through RJ, which unfolds across different stages of the restorative process.

Third, the film expands the meaning of community beyond those physically participating in restorative encounters. Through facilitators, intimate partners, and family members, restoration is portrayed as embedded within broader communities of care that prepare, sustain, and accompany participants before, during, and after the restorative process. The film therefore suggests that RJ extends beyond formal legal encounters by mobilizing a wider constellation of social actors whose caring practices become functionally integrated into the restorative process. Restoration thus depends on the institutional recognition and coordination of relationships that lie beyond the boundaries of formal justice institutions. At the same time, the film shows that communities of care may also reproduce silence, exclusion, and harm, reminding viewers that community itself can become an object of restoration.

Finally, the film speaks directly to contemporary debates concerning the role of community within RJ. Willis (2016) argues that community should be understood less as a pre-existing social entity than as something continuously negotiated through restorative practices. Likewise, Rosenblatt (2014) warns against treating community as an unproblematic concept, emphasizing instead the diverse forms of participation through which RJ is enacted. The film *All Your Faces* reflects these insights by portraying community as an ongoing relational accomplishment sustained through ritual, mutual recognition, and the ordinary practices of everyday life. In doing so, it offers a compelling sociological reflection on the relational foundations of RJ and on the possibilities, and limits, of rebuilding human relationships after harm.

More broadly, this article also demonstrates the value of film analysis for socio-legal research. Films participate in constructing cultural understandings of law, justice, responsibility, punishment, and restoration. They show the normative assumptions through which legal institutions acquire legitimacy and public meaning, offering insight into the legal culture surrounding RJ and the ways in which alternative models of justice become socially imaginable. Film analysis therefore complements empirical socio-legal research by revealing how legal ideas are culturally produced, circulated, and legitimized beyond formal legal settings. *All Your Faces* constructs a particular vision of what restoration, accountability, and community might look like. Examining these representations complement empirical research by revealing the symbolic and normative assumptions through which RJ acquires meaning within contemporary society.

Ultimately, the focus on a single film necessarily defines the analytical scope of this study. We examined *All Your Faces* as an in-depth case through which to explore how community is culturally represented and made meaningful within RJ. Our aim was to develop a situated interpretation of this particular cinematic representation rather than to generalize across films depicting restorative practices. A comparative analysis of multiple films could extend this approach by examining how different cinematic, cultural, and legal contexts construct the relationship between RJ and community. Such research could identify recurring representations as well as context-specific understandings of community, thereby complementing the in-depth analysis developed in the present article.

## Data Availability

The data supporting the conclusions of this article will be made available by the authors upon reasonable request.

## Footnotes

1 Although referred to here as a film, *All Your Faces* is more accurately described as a docufiction (Loncan, 2024), a genre that recreates real-life situations through fictional narrative. The film features professional actors, several of whom are members of the permanent ensemble of the Comédie Française in Paris.

2 The list is available at: https://www.euforumrj.org/films-and-documentaries. The announcement of the film screening can be found at: https://www.euforumrj.org/events/film-screening-je-verrai-toujours-vos-visages. The EFRJ presents *All your faces* by stating that “In 2023, a new film on restorative justice was released in France. It brought restorative justice in the centre of public attention, making it widely known to a public completely unaware of it previously.”

3 The first author watched the film on BBC I-player while she was living in England. The second author bought the movie on sooner.fr. The first author does not speak nor understand French, so she watched the movie in French with English subtitles. The second author is fluid in French, so she watched the movie in original language without subtitles, which was beneficial in preserving authentic meanings during the analysis.
